# The RsRlpA Effector Is a Protease Inhibitor Promoting *Rhizoctonia solani* Virulence through Suppression of the Hypersensitive Response

**DOI:** 10.3390/ijms21218070

**Published:** 2020-10-29

**Authors:** Spyridoula N. Charova, Fredrik Dölfors, Louise Holmquist, Panagiotis N. Moschou, Christina Dixelius, Georgios Tzelepis

**Affiliations:** 1 Institute of Molecular Biology and Biotechnology Foundation of Research and Technology-HELLAS, GR-70013 Heraklion, Crete, Greece; charova@imbb.forth.gr (S.N.C.); panagiotis.moschou@slu.se (P.N.M.); 2 Department of Biology, University of Crete, Voutes University Campus, P.O. Box 2208, GR-70013 Heraklion, Crete, Greece; 3 Department of Plant Biology, Swedish University of Agricultural Sciences, Uppsala BioCenter, Linnean Center for Plant Biology, P.O. Box 7080, S-75007 Uppsala, Sweden; fredrik.dolfors@slu.se (F.D.); christina.dixelius@slu.se (C.D.); 4 MariboHilleshög Research AB, Säbyholmsvägen 24, S-26191 Landskrona, Sweden; louise.holmquist@maribohilleshog.com; 5 Department of Forest Mycology and Plant Pathology, Swedish University of Agricultural Sciences, Uppsala BioCenter, Box 7026, SE-750 07 Uppsala, Sweden

**Keywords:** basidiomycete, biotrophy, effector, cathepsin, hypersensitive response, papain inhibitor, protease, soil-borne pathogen, sugar beet

## Abstract

*Rhizoctonia solani* (Rs) is a soil-borne pathogen with a broad host range. This pathogen incites a wide range of disease symptoms. Knowledge regarding its infection process is fragmented, a typical feature for basidiomycetes. In this study, we aimed at identifying potential fungal effectors and their function. From a group of 11 predicted single gene effectors, a rare lipoprotein A (RsRlpA), from a strain attacking sugar beet was analyzed. The *RsRlpA* gene was highly induced upon early-stage infection of sugar beet seedlings, and heterologous expression in *Cercospora beticola* demonstrated involvement in virulence. It was also able to suppress the hypersensitive response (HR) induced by the Avr4/Cf4 complex in transgenic *Nicotiana benthamiana* plants and functioned as an active protease inhibitor able to suppress Reactive Oxygen Species (ROS) burst. This effector contains a double-psi beta-barrel (DPBB) fold domain, and a conserved serine at position 120 in the DPBB fold domain was found to be crucial for HR suppression. Overall, *R. solani* seems to be capable of inducing an initial biotrophic stage upon infection, suppressing basal immune responses, followed by a switch to necrotrophic growth. However, regulatory mechanisms between the different lifestyles are still unknown.

## 1. Introduction

Plants are constantly exposed to vast numbers of microorganisms and insects. Survival depends on efficient recognition of the intruders and the timely activation of the local and systemic defense machinery. To succeed, plants have evolved two main strategies to detect pathogens [1,2]. The first defense level resides on the external face of a plant cell and includes conserved secreted microbial elicitors, called pathogen-associated molecular patterns (PAMPs), which are recognized by plant receptor proteins or pattern recognition receptors (PRRs) [3]. PAMPs are typically essential components of all classes of pathogens, such as the bacterial flagellin or the fungal chitin. Plants also respond to endogenous molecules released by pathogen invasion, such as cell wall fragments. Altogether, stimulation of PRRs leads to PAMP-triggered immunity (PTI). The second level of defense involves recognition of pathogen virulence molecules, termed effectors, by intracellular nucleotide-binding–leucine-rich repeat receptor genes, and most of them function as resistance (*R*) genes. This recognition induces effector-triggered plant immunity (ETI). ETI has co-evolved together with specific pathogen genotypes leading to strain or race-specific interactions, which culminates in local and strong hypersensitive response (HR) of plant-host cells restricting pathogen spread. Diversification of ETI receptors and pathogen effectors both within and between species is the norm, whereas the function of some PRRs is conserved widely across plant families [4]. Generally, PTI and ETI give similar overall responses, such as HR, although ETI is qualitatively stronger and much faster [5].

Soil contains millions of organisms and the nutrient-rich root exudates region attracts a myriad of invaders, including pathogens. Soil-borne pathogens represent an understudied category of plant pathogens, much due to their choice of the environment which is complex to monitor and control. The fungus *Rhizoctonia solani* (in Greek “root killer”) has a wide host range and causes the damping-off disease in seedlings, although plants in all ages can be infected [6]. The disease was first reported in 1858 by Kühn and has a growing impact due to warmer climate conditions. Despite knowledge of *R. solani* for more than 160 years many questions remain to be answered on its lifecycle and infection processes. This basidiomycete does not form any asexual spores, while the sexual stage (teleomorph: *Thanatephorus cucumeris*) is extremely rare. Restring structures (microsclerotia) can be formed, which can survive in the soil or plant debris for long periods before new rounds of infection [7,8]. *Rhizoctonia solani* isolates vary in morphology and genetics and they are grouped in different anastomosis groups (AGs) based on hyphal anastomosis reactions [9]. Isolates that belong to the AG2-2IIIB group are more aggressive against sugar beets and cause root and crown rot, damping off or foliar blight [10].

The majority of effectors, which have been characterized in *R. solani* so far, are necrosis-inducing ones [11,12,13,14]. In this work, we aimed to identify novel effectors for the *R. solani*-sugar beet interaction. We prioritized a predicted singleton from the genome dataset of the *R. solani* AG2-2IIIB strain [15] encoding a rare lipoprotein A (*RlpA*) gene, henceforth *RsRlpA.* This protein previously has been reported in bacterial strains, but its exact function is still unknown [16]. We found that RsRlpA was able to suppress PTI-related HR induced by the Avr4/Cf4 complex. Avr4 is a chitin-binding effector recognized by the Cf4 receptor in resistant tomato plants leading to strong HR [17,18]. It is also an active protease inhibitor, interacting with a plant cathepsin. Taken together, this study shows that *R. solani* deploys effectors other than necrosis-inducing ones, indicating that *R. solani* relies on an initial biotrophic stage to establish a successful infection, followed by extensive necrotrophic growth when plant tissue is heavily colonized.

## 2. Results

### 2.1. The RsRlpA Contains a DPBB Fold and Is Highly Induced upon Early Infection

**Figure 1:**
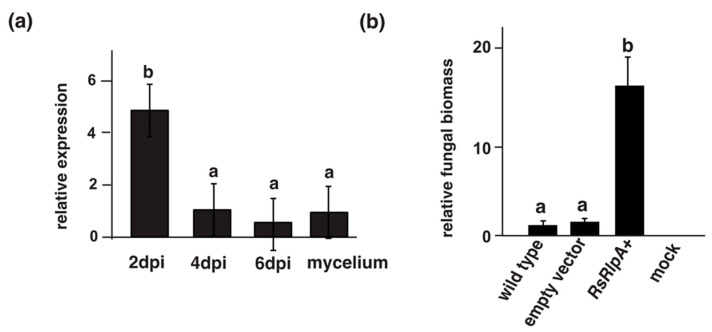
Analysis of the RsRlpA candidate effector. (**a**) Transcription patterns of the *RsRplA* gene upon sugar beet infection. *Rhizoctonia solani* mycelia was grown in potato dextrose broth (PDB) medium and used as control for comparison. The *G3PDH* gene was used as internal standard. Error bars represent SD based on at least three biological replicates. Different letters (**a**,**b**) indicate statistically significant differences according to Tukey’s test (*p* value < 0.05). (**b**) Fungal biomass quantification upon infection of sugar beet leaves with *Cercospora beticola* wild type, empty vector and *RsRlpA+* overexpression strains, while inoculation with H_2_0 (mock) was used a negative control. For qPCR, the *C. beticola* actin (*act*) gene was used. Data were normalized with the elongation factor gene (*elf-1*) from *Beta vulgaris*. Data show the average of three independent overexpression strains and three biological replicates. Different letters (**a**,**b**) indicate significant differences according to Tukey’s test (*p* < 0.05) and error bars represent SD.

Genome analysis of *R. solani* AG2-2IIIB (Rs) revealed 126 predicted, secreted and cysteine-rich proteins [15]. Among them, only 61 were shorter than 400 amino acids and cysteine-rich, and thus considered as candidate effectors [19,20]. Eleven remaining single-gene candidates were predicted, including the *RsRlpA* (*RSOLAG22IIIB_08473*) gene. We first monitored the transcript levels in a time-course of sugar beet plantlets grown in *R. solani* infested soil. The *RsRlpA* gene was highly induced 2 days post inoculation (dpi) compared to 4 and 6 dpi, indicating a role in the early infection process (Figure 1a). Next, the protein structure of RsRplA was investigated. This gene encodes a putative rare lipoprotein A (RlpA)-like protein containing a conserved region that has a double-psi beta-barrel (DPBB) fold (IPR009009) (Figure S1). Structure analyses revealed 37% homology to a papain inhibitor from *Streptomyces* species, 35% to a cellulose-binding protein from *Clavibacter* species and 29% to kiwellin, a protein present in plant cell walls.

### 2.2. Heterologous Expression of the RsRlpA Promotes Fungal Infection

**Figure 2:**
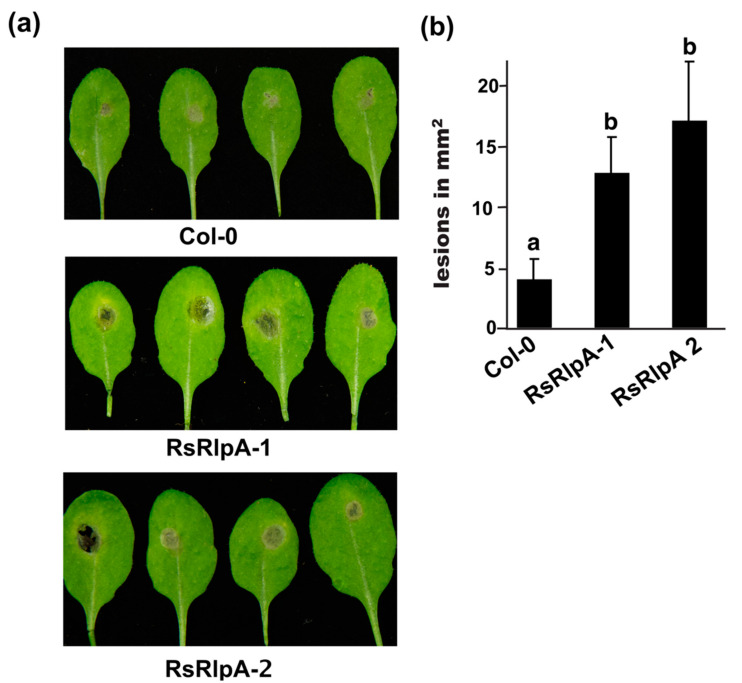
Expression of *RsRlpA* in *Arabidopsis thaliana* promotes fungal infection. (**a**) Symptoms of *A. thaliana* leaves from wild type (Col-0) and overexpression *RsRplA* lines after infection with *Botrytis cinerea* conidia. Images taken 3 dpi. (**b**) Lesion area on *A. thaliana* leaves infected with *B. cinerea* 3 dpi. Different letters (**a**,**b**) indicate significant differences according to Tukey’s test (*p* < 0.05). Error bars represent SD of four biological replicates, containing four infected leaves from four plants.

To investigate the role of RsRlpA in virulence, we chose the sugar beet ascomycete pathogen *Cercospora beticola* for heterologous gene analysis, since *R. solani* is not amenable to any kind of molecular editing. Reverse transcription (RT) PCR analysis confirmed the expression of *RsRlpA*+ strains (Figure S2a). Confocal microscopy demonstrated active GFP in overexpression and empty vector strains (Figure S3). The fungal biomass in *RsRlpA+* infected plant tissues was significantly increased further supporting the importance of *RsRlpA* in the infection process (Figure 1b). No differences in the size of necrotic lesions were observed among *C. beticola* wild type (WT) and the *RsRplA+* strains. (Figure S4). The role of *RsRlpA* in virulence was also investigated in transgenic *Arabidopsis thaliana* plants (Col-0) harboring a *35S*:*RsRplA* construct. Phenotypic analysis of these lines displayed no differences as compared to WT plants, despite the high expression of *RsRplA* (Figure S2b). Potential responses of these overexpression lines to *Botrytis cinerea* were tested. Infected leaves showed more severe symptoms compared to WT (Figure 2a,b) suggesting a non-specific recognition of the *RsRplA* in plant-fungal interactions.

### 2.3. The RsRplA is Localized to the Cell Periphery and Suppresses Hypersensitive Response Induced by the Avr4/Cf4 Complex

**Figure 3:**
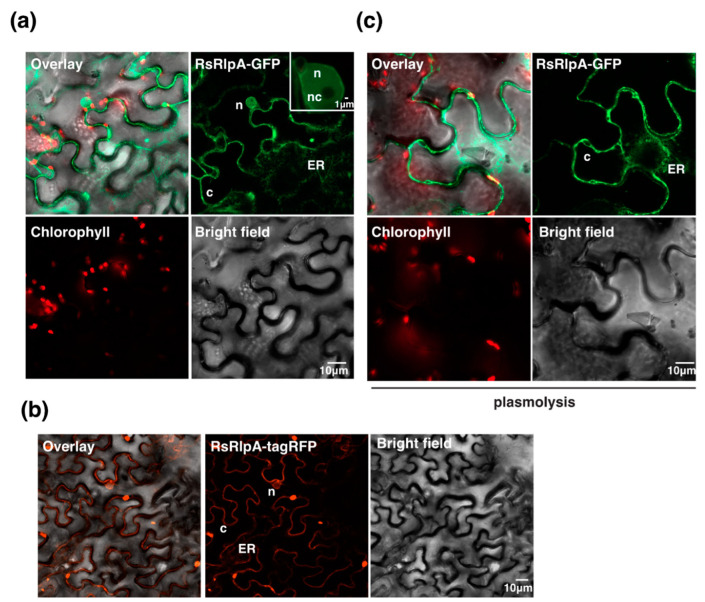
The RsRplA effector is localized to plant cell periphery. Live-cell imaging of (**a**) C-terminal GFP-tagged or (**b**) tagRFP-tagged RsRlpA in Agro-infiltrated *N. benthamiana* leaves. The localization was monitored with a laser-scanning confocal microscope with a sequential scanning mode 48 h post infiltration. The GFP and the chlorophyll were excited at 488 nm. GFP (green) and chlorophyll (red) fluorescent signals were collected at 505–525 and 680–700 nm, respectively. (**c**) Plasmolysis was incited by using 1M mannitol for 30 min. The tagRFP was excited at 558 nm and collected at 545–620 nm. c: cytoplasm, ER: endoplasmic reticulum, n: nucleoplasm, nc: nucleolus.

To monitor the localization of RsRlpA in plant cells, a construct was made with GFP at the C-terminus, keeping the signal peptide intact, and transiently expressed in *Nicotiana benthamiana* plants. The RsRplA protein was accumulated in the cell periphery and nucleoplasm (Figure 3a). The RsRlpA was also tagged with RFP at the C-terminus. Our data showed localization in the cell periphery similar to the GFP-tagged variants, but no localization to the nucleus was observed (Figure 3b), indicating that it was most likely free GFP that localized to the nucleoplasm. Treatment of leaves with mannitol (plasmolysis) confirmed the localization to the cell periphery (Figure 3c).

**Figure 4:**
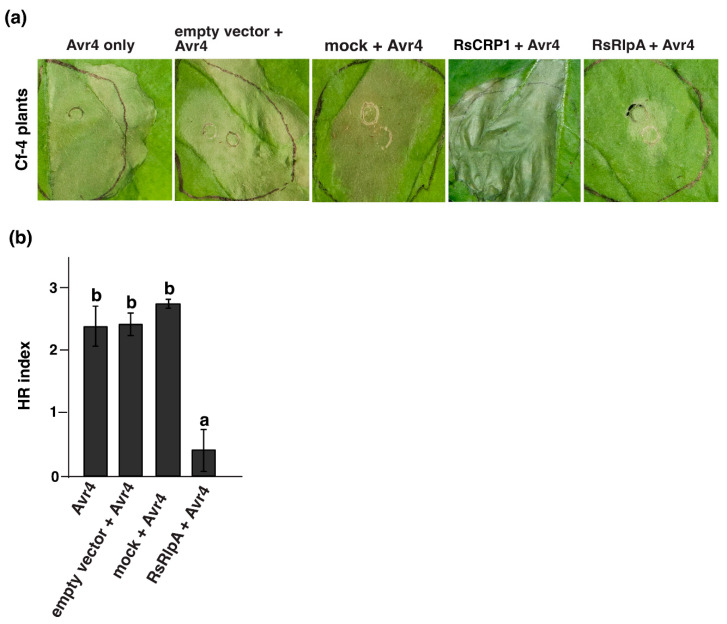
The RsRplA effector suppresses the Avr4-mediated hypersensitive response (HR). (**a**) HR suppression assay by the RsRplA on *N. benthamiana* expressing the Avr4/Cf4 complex. (**b**) HR index-scale from 0–3 with “0” indicates no symptoms and “3” severe symptoms on *N. benthamiana*. Leaves were Agro-infiltrated first with the RsRlpA effector driven by the 35S promoter and HR challenged 24hpi with the Avr4 effector. Agro-infiltration with: empty vector, induction buffer (mock) or the RsCRP1 candidate effector protein were used as negative controls. Images taken 3dpi. Different letters (**a**,**b**) indicate significant differences according to Tukey’s test (*p* < 0.05). Error bars represent SD of samples of two Agro-infiltrated leaves from eight plants.

Firstly, we checked whether RsRplA could induce necrosis. In contrast to earlier studies [11,12,13,14], transient expression of this protein in *N. benthamiana* did not induce cell death (Figure S5). Furthermore, it is known that many pathogens secrete effectors function as HR suppressors [19,20]. For that reason, the ability of RsRlpA to suppress HR was analyzed in the Avr4/Cf4 complex [17,18]. In leaf areas where the RsRlpA had been previously Agro-infiltrated, significantly reduced HR was observed compared to the area where only the Avr4 was expressed (Figure 4a,b). In parallel, potential suppression of ETI-triggered HR was checked in *N. bethamiana* infected with *Pseudomonas syringae* pv. *tomato* DC3000. This bacterial strain secretes effector proteins to the hosts through the type III secretion system, recognized by *N. benthamiana* R proteins, leading to a strong HR [21]. We found that RsRplA was not able to suppress HR induced by this bacterial strain (Figure S5). Taken together, these data suggest that RsRlpA is only involved in PTI responses.

### 2.4. The RsRlpA is an Active Protease Inhibitor Suppressing Oxidative Burst

**Figure 5:**
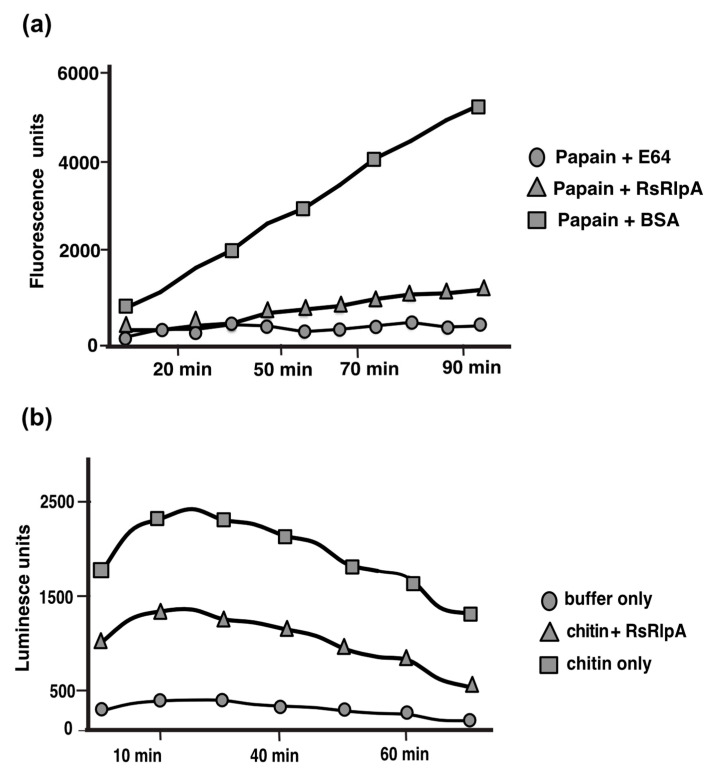
The RsRplA is a protease inhibitor suppressing ROS burst. (**a**) Protease inhibition assay using 10 μM of purified RsRlpA protein mixed with the Z-Phe-Arg-NMec substrate and 5 μM papain. The protease inhibitor E64 and BSA protein were used as positive and negative controls, respectively. (**b**) Chitin-induced oxidative (ROS) burst assay in *N. benthamiana* leaves. Production of ROS was determined using luminol-dependent chemiluminescence. Leaf discs were treated with chitin, 10 μΜ RsRplA protein, while only buffer was used as a negative control. Eight biological replicates were run in both assays.

As mentioned before, the predicted 3D structure of RsRlpA displayed homology to papain-like inhibitors. Thus, we investigated whether this protein was able to block proteolytic activity. To this end, we expressed RsRplA in *E. coli* cells and nanoLC-MS/MS analysis confirmed the correct amino acid sequence of this protein. The inhibitory activity of RsRlpA was examined against papain, a common plant protease from the papaya plant. The Z-Phe-Arg-NMec substrate was used, which upon cleavage emits fluorescence which can be quantified fluorometrically. The RsRlpA was able to block papain proteolytic activity, similar to the N[N-L-3-trans-carboxyoxirane-2-carbonyl-L-leucyl]-agmatine (E-64) inhibitor, a well-known protease inhibitor, used as a positive control (Figure 5a). To clarify whether RsRlpA was also able to suppress oxidative burst, a luminol-based assay was used. ROS production was significantly reduced on leaves which previously had been treated with the RsRplA protein compared to non-treated leaves (Figure 5b). These data suggest that suppression of HR is possibly attributed to the reduction in ROS burst.

### 2.5. The RsRplA Associates with a Plant Cathepsin

**Figure 6:**
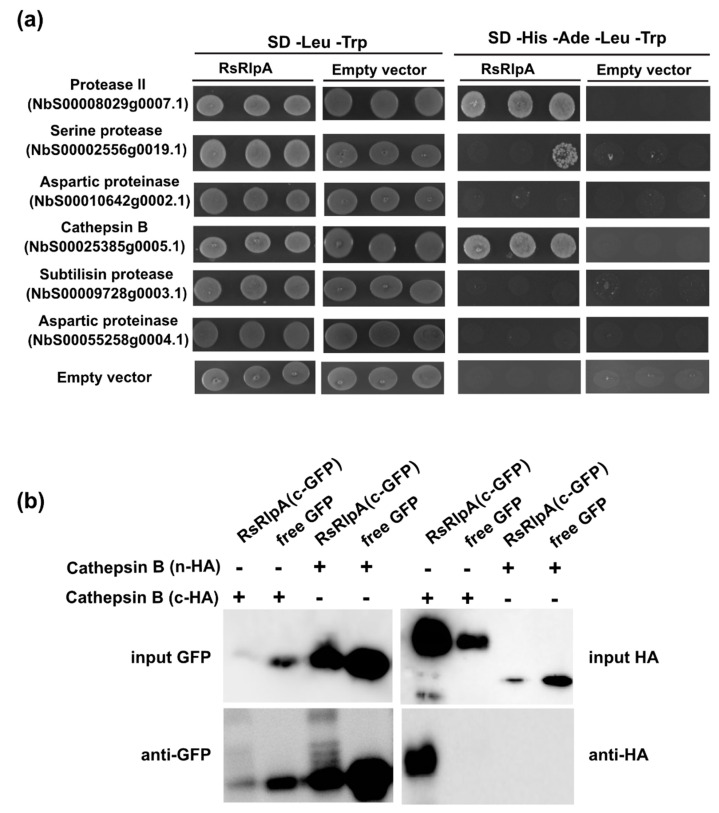
The RsRplA interacts with a plant cathepsin. (**a**) Pairwise yeast-two-hybrid analysis between RsRlpA (used as a prey in pGADT7 vector) and potentially interacting plant proteases (used as baits in pGBKT7 vector). Growth of yeast cells on SD-4 (-His, -Ade, -Leu, -Trp) selective media represents protein–protein interaction and growth on SD-2 (-Leu, -Trp) media confirms yeast transformation. Yeasts transformed with the empty vectors were used as negative controls. (**b**) Co-immunoprecipitation (co-IP) assay between the GFP-tagged RsRlpA and HA-tagged cathepsin, transiently co-expressed in *N. benthamiana* and pull-downed using the GFP-trap agarose magnetic beads.

To study whether RsRlpA interacts with plant proteases, this protein was tagged with GFP, immunopurified from *N. benthamiana* and interacting proteins were analyzed by MS/MS. Among the candidate proteases, six were selected for pairwise yeast-two-hybrid analysis (Table S1). Using RsRlpA as a prey and plant proteases as baits, we identified two proteases potentially interacting with the RsRlpA; protease II (NbS00008029g0007.1) and cathepsin B-like cysteine proteinase (NbS00025385g0005.1) (Figure 6a). Structure analysis of cathepsin B-like revealed that it is a papain-like C1A cysteine protease, containing a signal peptide and a cathepsin pro-peptide inhibitor domain (I29).

Previously, plant cathepsins have been reported to be involved in different processes such as programmed cell death and autophagy [22,23]. To test RsRlpA-cathepsin B interaction a co-immunoprecipitation assay was conducted. Cathepsin B was tagged with the HA epitope (either in C or N terminus) and transiently co-expressed with the GFP-tagged RsRlpA in *N. benthamiana*. Our results demonstrated that the C-HA tagged cathepsin B-like could co-immunoprecipitate with RsRlpA (Figure 6b), indicating that this interaction leads to HR suppression.

### 2.6. The Ser120 is Involved in RsRplA Ability to Suppress Cell Death

**Figure 7:**
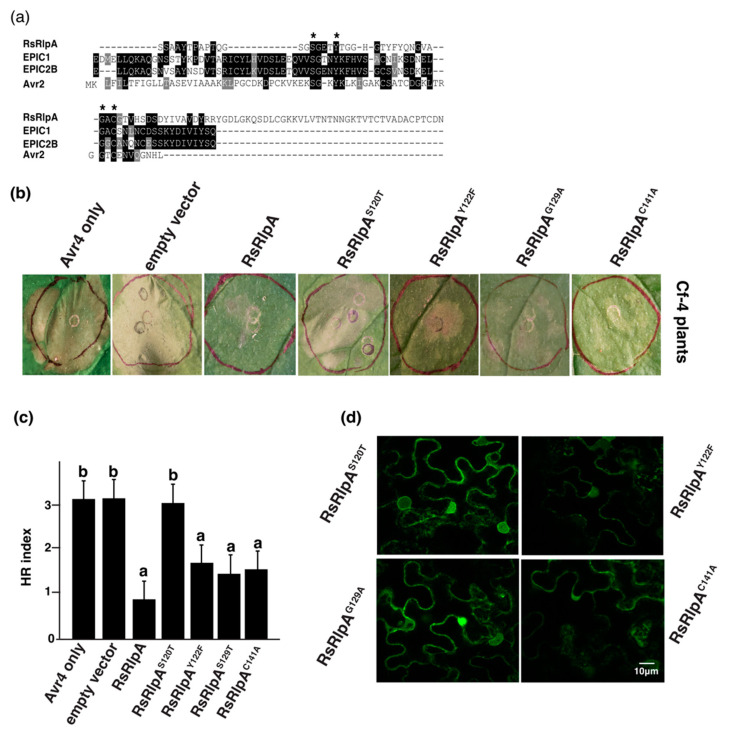
Serine 120 in the RsRplA protein is essential for suppression of Avr4-mediated HR. (**a**) Alignment among the RsRplA amino acid sequence and protease inhibitors from *Phytophthora infestans* (EPIC1 and EPIC2) and *Cladosporium fulvum* (Avr2) conducted by Clustal X algorithm. Asterisks indicate identical amino acids. (**b**) HR suppression assay by the RsRplA mutants on *N. benthamiana* plants expressing the Cf4/Avr4 complex. (**c**). HR scale from 0–3 where ‘’0‘’ indicates no symptoms and “3” severe symptoms in *N. benthamiana*. Leaves were Agro-infiltrated first with the RsRlpA or mutants and HR challenged 24hpi with the Avr4 effector. Empty vector was used as a negative control. Images taken 3dpi. Different letters (**a**,**b**) indicate significant differences according to Tukey’s test (*p* < 0.05). Error bars represent SD of eight plants, each containing two Agro-infiltrated leaves. (**d**) Live-cell imaging of C-terminal GFP-tagged RsRlpA mutants in Agro-infiltrated *N. benthamiana* leaves. The localization was monitored with a laser-scanning with a sequential scanning mode 48 hrs post infiltration. The GFP was excited at 488 nm and collected at 505–525 nm.

Amino acid sequences among known effector proteins function as protease inhibitors were then compared. The EPIC1 and EPIC2 from *Phytophthora infestans*, which target the *Phytophthora* Inhibited Protease 1 (PIP1), a papain-like cysteine protease (PLCP) from tomato [24] and the Avr2 effector from the hemibiotrophic pathogen *C. fulvum*, which inhibits the tomato Rcr3 protease [25] were aligned with RsRlpA. We found that S120, Y122, G129 and C141 were identical between these four effectors (Figure 7a). To evaluate the importance of the conserved amino acids, point mutations were created. In the case of RsRlpA, these four amino acids were located in the DPBB fold. When monitoring the impact of the RsRlpA^S120T^ mutant, it failed to suppress HR induced by the Avr4/Cf4 complex (Figure 7b,c). No visible effects were seen from the other three mutants (Figure 7b,c). The in-planta localization and protein stability of the four RsRlpA mutants were unaltered (Figure 7d).

## 3. Discussion

*Rhizoctonia solani* is a soil-borne plant pathogen with an almost unknown infection cycle. Here we have presented data suggesting that *R. solani* is a potential hemibiotrophic pathogen since it has capacity to generate features of a short biotrophic stage. Mechanistic studies of selected genes in *R. solani* is complicated since this species is not amenable to any genetic modification. Thus, the employment of tools and strategies from other plant-pathogen systems is the only way forward to generate new information.

Here we followed-up data generated in the genome sequencing of the sugar beet *R. solani* isolate AG2–2IIIB, known also to cause disease in maize [26]. A predicted unigene effector candidate encoding a rare lipoprotein A (RlpA)-like protein was chosen for further analysis using multiple approaches. The RsRlpA protein contains a DPBB fold with six β-barrels identified previously in various protein super-families, such as expansins, dehydrogenases, endo-glucanases, and in papain-like inhibitors [27,28]. For example, in the rice blast pathogen *Magnaporthe oryzae*, induction of a RlpA-like gene (*MGG_07556.6*) was identified at the early biotrophic stage of rice infection [29]. These data are in agreement with the *RsRplA* transcription patterns, indicating an involvement of this effector at the early stages of the infection. RlpAs have also been identified in the secretomes of virulent *Pyrenophora teres* f. *teres* isolates and in ectomycorrhizal species causing brown and white rot [30,31]. In bacteria, the RlpAs are involved in morphogenesis, cell division and peptidoglycan metabolism [32,33]. Despite the rather frequent incidence among various microorganisms, not much is known about their function in a plant defense context. Cysteine protease inhibitors are also present in plants, for example CaPR4c in pepper that interacts with the AvrBsT effector from *Xanthomonas campestris* [34]. CaPR4c is required for HR and is localized at the plant plasma membrane during cell death. Thus, this type of responses seems to be a common trans-kingdom event.

The predicted protein structure of RsRplA showed homology to papain-like inhibitors. Papain-like cysteine proteases (PLCPs) are involved in numerous plant immune responses against invading pathogens, such as induction of cell death or function as co-receptors for R proteins [35,36,37]. Their precise roles in plant immunity remain to be elucidated. It is speculated that they release damage-associated molecular patterns (DAMPs) which can induce defense mechanisms [38]. Hence, PLCPs are obvious targets for particularly biotrophic pathogens to suppress such responses. Here we found that RsRplA is an active protease inhibitor suppressing papain proteolytic activity and induction of HR by interacting with a plant cathepsin. Effectors inhibiting PLCPs have been previously reported in biotrophic and hemibiotrophic plant pathogens [39,40,41] and the roles of these inhibitors in the suppression of HR have been demonstrated [42,43].

**Figure 8:**
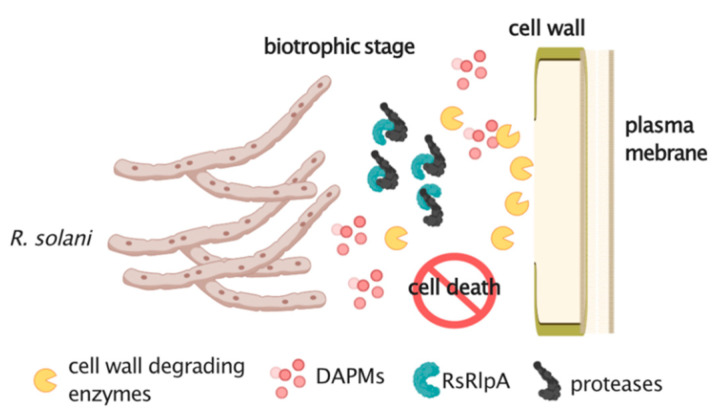
Proposed function of the RsRplA effector during plant infection. Carbohydrate active enzymes degrade the plant cell wall and produce damage-associated molecular patterns (DAMPs) able to induce pattern-triggered immunity (PTI). The RsRplA effector could be deployed by *R. solani* to perturb protease activity suppressing hypersensitive response during an initial biotrophic stage. This is followed by activity of cell wall degrading enzymes leading to the onset of the necrotrophic stage. Figure created by the Biorender software.

Lifestyles are rarely a strict phenomenon. Changes between different forms or ways to invade or somehow associate with a host plant are far from rare [44]. Based on our studies on the *R. solani* sugar beet strain AG2-2IIIB, we propose the following model of its different modes of lifestyle (Figure 8). This basidiomycete spends most of its time in soil and infected debris. It has a large repertoire of carbohydrate-active enzyme (CAZy) encoding genes in its genome suitable for cell wall degradation and possible saprophytic survival [25]. These enzymes can generate DAMPs able to induce PTI. At the same time, *R. solani* possibly deploys effectors to suppress basal host defense responses, such as suppression of programmed cell death, in order to establish a successful infection. These results, in combination with our previous studies, showing that *R. solani* produces a chitin-binding LysM effector perturbing chitin-induced immunity, supporting the preference of this pathogen to living cells (biotrophic state) at least at the early stages of infection [45]. Intriguingly, in sugar beet, both major latex proteins (MLPs) and NBS-LRR encoding *R* genes contribute to *R. solani* defense, suggesting that multiple fungal invasion and defense strategies are employed [46].

Progression of the pathogen growth and host colonization leads to the sugar beet root and crown rot. This late necrotrophic phase of the infection is most likely dominated by the CAZYs and necrosis-inducing effectors encoded by the fungus. Signals or environmental conditions that determine the switches between the different growth and infection strategies of *R. solani* remain to be identified.

## 4. Materials and Methods

### 4.1. Fungal Isolates and Growth Conditions

The *R. solani* AG2-2IIIB isolate BBA 69,670 (DSM 101808), originating from sugar beet plants grown in Bavaria, Germany, was used in the current study [26]. Soil was infested with *R. solani* using media containing perlite, corn flour and water in 1:1:5 ratio. *Cercospora beticola* isolate Ty1, isolated from infected sugar beet leaves grown in Germany, was kept on potato dextrose agar (PDA, DB Difco, Franklin Lakes, NJ, USA) plates at 22 °C in darkness and sporulation was induced on tomato growth extract medium [47]. *Botrytis cinerea* strain B05.10 [48] was kept on PDA (DB Difco, Franklin Lakes, NJ, USA) and sporulation was induced by 0.5 M NaCl under continuous blue light.

### 4.2. RNA Isolation and qRT-PCR Analysis

Three-week-old sugar beet plantlets were transplanted into soil infested by the *R. solani* as described previously [45]. RNA from plantlets 2, 4, and 6 days post infestation (dpi) and *R. solani* mycelia, grown in potato dextrose broth (PDB, DB Difco, Franklin Lakes, NJ, USA) and used as a control, was extracted using the RNeasy Plant Mini Kit (Qiagen, Hilden, Germany), according to manufacturer’s instructions. qRT-PCR was carried out as previously described [49]. Primers are listed in Table S2. Data were normalized to *R. solani G3PDH* (*RSOLAG22IIIB_07022*) transcript levels [50] and relative expression values were calculated according to the 2^−ΔΔCt^ method [51]. At least three biological replicates were used.

### 4.3. Construction of Cercospora Beticola Transgenic Strains and Infection Assays

For the construction of *C. beticola* transgenic overexpression strains, the In-Fusion cloning technology was used (Takara, Kusatsu, Japan). The *RsRplA* gene from *R. solani* cDNA was amplified using the primers listed in Table S2. The gene fragment was ligated to the pRFHUE-eGFP vector [52] and transformed to *C. beticola* using an *Agrobacterium*-mediated protocol [53]. The expression of *RsRlpA* gene in transgenic strains was investigated by reverse transcription PCR and confocal microscopy.

Leaves of three-week-old sugar beet plantlets were infected with 10^6^ conidia/mL derived from *C. beticola* (WT), overexpression strains (*RsRlpA+*) and strains where only the empty vector was expressed as previously described [45]. Mock inoculation with H_2_0 was used as a negative control. The fungal DNA biomass was quantified in infected leaves 7 dpi using quantitative real-time PCR. Total genomic DNA was extracted from infected leaves using a CTAB-mediated protocol [54]. Fungal DNA was quantified using the *C. beticola* actin (*act*) reference gene and normalized with *B. vulgaris* elongation factor (*elf-1*) gene. Primers are listed in Table S2.

### 4.4. Transgenic Arabidopsis Plants and Pathogenicity Assays

The *RsRlpA* effector sequence was amplified from *R. solani* cDNA using the Phusion Taq polymerase (Thermo Fisher Scientific, Waltham, MA, USA), cloned into pENTR/D-TOPO Gateway vector (Thermo Fisher Scientific, Waltham, MA, USA). The orientation and integrity of the insertion were confirmed by DNA sequencing (Macrogen Inc., Seoul, South Korea). Clones entered the pGWB602 binary vector and transformed to *Agrobacterium tumefaciens* strain C58 cells, followed by transformation to *Arabidopsis* Col-0 using the floral dip method as previously described [55]. Expression of the *RsRlpA* in transgenic lines was confirmed by RT-PCR. Rosette leaves of three-week-old *Arabidopsis* plants grown on short-day conditions (8 h light/16 h dark) at 22 °C/17 °C, were inoculated with 10^6^/mL conidia of *B. cinerea* and symptoms were evaluated after three days. Four biological replicates for each transgenic line were used. Each sample comprised four plants and four infected leaves per plant.

### 4.5. Effector Subcellular Localization and Hypersensitive Response Assays

Using the *RsRplA*:pENTR/D-TOPO clone, the *RsRplA* gene entered to the pGWB605 or pGWB660 binary vectors, tagged with C-terminus GFP or tagRFP fluorescence proteins, respectively. Overnight cultures of *A. tumefaciens* (C58) harboring the 35S:*RsRplA* constructs were used for Agro-infiltration on 4-week old *N. benthamiana* leaves [56]. The subcellular localization of the RsRlpA effector was monitored by using a Zeiss LSM 800 confocal microscope. The GFP was excited at 488 nm and collected at 505–525 nm, and tagRFP was excited at 558 nm and collected at 545–620 nm.

For the HR suppression assay, the *RsRlpA* gene was entered to the pGWB602 binary vector and transiently expressed in *N. benthamiana* plants (grown under 17 h light/7 h dark at 23 °C) harboring the Cf-4 receptor protein from tomato plants. The condition was: OD_600_ = 0.5 in 10 mM MES, 10 mM MgCl_2_ and 150 μM acetosyringone. The HR was triggered 24 hrs after RsRplA Agro-infiltration with the *Cladosporium fulvum* Avr4 effector protein at OD_600_ = 0.03. The HR suppression ability of RsRlpA was also investigated in *N. benthamiana* wild type plants upon infection with *P. syringae* pv*. tomato* DC3000 as described previously [57]. Agro-infiltration with empty vector, only induction buffer (mock) and the *R. solani* RsCRP1 (RSOLAG22IIIB_02432) effector candidate were used as negative controls.

For single point mutation, the procedure by Zheng et al. [58] was applied using primers for site-directed mutagenesis listed in Table S2. PCR products treated with the *Dpn*I restriction enzyme, ligated to pGWB602 vector and transformed to *A. tumefaciens* C58 cells. The HR suppression assay for the four mutants (RsRlpA^S120T^, RsRlpA^Y122F^, RsRlpA^G129A^ and RsRplA^C141A^) was conducted as described above. In total, eight four-week-old plants were Agro-infiltrated and two upper leaves in each plant were used. HR response was evaluated using a scale from 0 to 3, ranging from no symptoms (0) to severe symptoms (3).

### 4.6. Prediction of Protein 3D Structure, Production and Purification

The presence of conserved domains in the RsRplA effector was tested using the SMART 6.0 protein analysis tool and InterProScan 5.0 [59,60]. Its 3D structure was predicted using the SWISS-MODEL [61] and the RaptorX servers [62]. For protein production, the entire *RsRlpA* gene was amplified from *R. solani* cDNA using primers in Table S2. PCR products were digested with *Nde*I and *Xho*I restriction enzymes (New England Biolabs, Ipswich, MA, USA), ligated to the pET26b(+) vector and transformed to *E. coli* SHuffle^®^ T7 cells (New England Biolabs, Ipswich, MA, USA). The orientation and integrity of the insertion were confirmed by DNA sequencing (Macrogen Inc., Seoul, South Korea). Protein production and purification were done as described previously [63]. Transformed cells were grown overnight in LB medium and protein production was induced by 1 mM IPTG (Thermo Fisher Scientific, Waltham, MA, USA) for 18 hrs at 18 °C. Then bacterial cells were precipitated and resuspended in 50 mL PBS buffer and homogenized using a cell press disruptor at 1.93 kbar. The supernatant was loaded onto HisPur cobalt chromatography cartridges (Thermo Fisher Scientific, Waltham, MA, USA) and eluted from the column using 150–300 mM imidazole. Protein identification was performed by denaturation with 8 M urea and MS/MS analysis (Proteome Factory AG, Berlin, Gernany). Before use, excess salt and imidazole were removed by eluting the protein through desalting spin columns (Thermo Fisher Scientific, Waltham, MA, USA).

### 4.7. Protease Inhibition and ROS Burst Suppression Assays

The protease inhibition assay was done as described previously [64] by measuring the enzymatic activity of Z-Phe-Arg-NMec. 10 μM of purified RsRlpA protein was mixed with 5 μM Z-Phe-Arg-NMec (Merck, Kenilworth, NJ, USA) and 5 μM papain (Sigma-Aldrich, St. Louis, MO, USA) in 850 μL of total reaction buffer (0.1 M buffer KH_2_PO_4_/Na_2_HPO_4_, pH 6.8; 4 mM cysteine; 1 mM Na_2_EDTA; 200 mM NaCl; 0.05% Brij 35). 10 μM of cysteine protease inhibitor E-64 (Sigma-Aldrich, St. Louis, MO, USA) or BSA protein were used as a positive and negative control, respectively. For the suppression of the oxidative burst of reactive oxygen species (ROS), a luminol-based protocol was used [65]. Briefly, leaf discs from *N. benthamiana* plants, treated with chitin (100 μL/mL), luminol (200 μM) (Sigma-Aldrich, St. Louis, MO, USA) and 10 μg/mL horseradish peroxidase (Sigma-Aldrich, St. Louis, MO, USA). The suppression of ROS was analyzed by using 10 μM RsRlpA protein and measuring the chemiluminescence levels.

### 4.8. Pull-Down and Mass Spectrometry

The RsRlpA protein, tagged with GFP at the C-terminal, was transiently expressed in *N. benthamiana* leaves, while free GFP was used as a negative control. Proteins were extracted using an extraction buffer containing 20 mM HEPES pH 6.8, 150 mM NaCl, 1 mM EDTA, 1 mM DTT, 0.5% Tween 20, 1 mM PMFS and proteases inhibitor cocktail (Roche) and pull-downed using the GFP-trap agarose magnetic beads (Chromotek, Munich, Germany). Samples proceeded for LC-ESI-MS/MS analysis at the Clinical Proteomics Mass Spectrometry facility, Karolinska Institute/Karolinska University Hospital/Science for Life Laboratory, Stockholm.

On-bead reduction, alkylation and digestion (trypsin, sequencing grade modified, Pierce) was performed, followed by SP3 peptide clean-up of the resulting supernatant [66]. Each sample was separated using a Thermo Scientific Dionex nano LC-system in a 3 hr 5–40% ACN gradient coupled to Thermo Scientific High Field QExactive. The software Proteome Discoverer vs. 1.4 including Sequest-Percolator for improved identification was used to search the *N. benthamiana* proteome database for protein identification, limited to a false discovery rate of 1%.

### 4.9. Protein-Protein Interaction

For the yeast-two-hybrid assay, full-length cDNA from the *RsRlpA* gene was ligated to the pGADT7 prey vector (Takara, Kusatsu, Japan) and plant proteases to the pGBKT7 bait plasmid (Takara, Kusatsu, Japan) and simultaneously transformed to the *Saccharomyces cerevisiae* AH109 strain. Transformations with empty vectors were used as negative controls. For co-immunoprecipitation assays, the RsRplA GFP-tagged protein and HA-tagged plant protease were transiently co-expressed in *N. benthamiana* and pull-downed as described above. GFP-tagged protein was detected using the B2 anti-GFP HRP-conjugated antibody (Santa Cruz Biotechnology, Dallas, TX, USA), and HA-tagged proteases were detected using the anti-HA peroxidase-conjugated antibody (Sigma-Aldrich, St. Louis, MO, USA), according to manufacturers’ instructions.

### 4.10. Statistical Analysis

Analysis of variance (ANOVA, one way) was conducted on gene expression and phenotypic data using a General Linear Model implemented in SPSS version 24 (IBM, Armonk, NY, USA). Pairwise comparisons were made using the Tukey’s test at the 95% significance level.

## Supplementary Materials

Supplementary Materials can be found at https://www.mdpi.com/1422-0067/21/21/8070/s1.

## Abbreviations

AG	Anastomosis group
DAMP	Damage-associated molecular patterns
CAZy	Carbohydrate-Active enZYmes
DPBB	Double psi beta barrel
DPI	Days post inoculation
ETI	Effector-triggered immunity
HR	Hypersensitive response
PAMPs	Pathogen-associated molecular patterns
PLCP	Papain-like cysteine protease
PRR	Pattern recognition receptors
PTI	Pattern-triggered immunity
RlpA	Rare lipoprotein A
ROS	Reactive oxygen species
