# Supplementary material for: The RsRlpA Effector Is a Protease Inhibitor Promoting Rhizoctonia solani Virulence through Suppression of the Hypersensitive Response
Source: Int J Mol Sci. 2020 Oct 29;21(21):8070. doi: 10.3390/ijms21218070 (PMC7662947; doi:10.3390/ijms21218070)
Supplement: Supplementary file 1 [file ijms-21-08070-s001.pdf]

Table S1. Candidate plant proteins putatively interact with the RsRplA effector

Protein ID	RsRplA-1	RsRplA-2	RsRplA-3	GFP-1	GFP-2	GFP-3
NbS00008029g0007.1 (Protease II Oligopeptidase B)	N/D	N/D	1,135E^7	N/D	N/D	N/D
NbS00002556g0019.1 (Serine protease)	9,007E^6	N/D	N/D	N/D	N/D	N/D
NbS00010642g0002.1 (Aspartic proteinase)	1,666E^7	N/D	N/D	N/D	N/D	N/D
NbS00025385g0005.1 (Cathepsin B)	2,867E^7	5,596E^6	N/D	6,866E^6	N/D	N/D
NbS00009728g0003.1 (Subtilisin protease)	N/D	N/D	1,228E^7	N/D	N/D	N/D
NbS00055258g0004.1 (Aspartic proteinase)	5,345E^6	N/D	N/D	N/D	N/D	N/D

*Values present the average peak area of the three most intense peptides including unique and non-unique ones after MS/MS analysis on *N. bethamiana* leaves transiently expressed the RsRplA effector. N/D: not detected.

Table S2. Primer sequences used in the current study

Primer name	Forward primer 5'-3'	Reverse primer 5'-3'	Annealing Tm	Reference
**qRT-PCR**				
RsRlpA-1	tcgtactctggctctggcaactct	cgcaggcaccggctactcc	58°C	This study
G3PDH	accgttatgggcttgtctttcctt	cccgcttggctggaatagtaacg	58°C	Chamoun et al 2015
***C. beticola* overexpression strain**				
RsRlpA+	gcagacatcacccgggatgttctctact gctgttgctgc	agtcctcagccccgggaccgatgactt gatagctaatgggaa	60°C	This study
**DNA quantification**				
Bv elf-1	cacgggaaatcaacgctcac	ttcatcggcacgggtatcag	58°C	Dölfors et al 2019
Cbactin	acatggctggtcgtgaatttg	tgtccgtcaggaagctcgta	58°C	This study
RsRlpA-2	caccaacaacggcaagactg	agagccttgaaagcaccta	65°C	This study
**Protein expression**				
RsRlpA (full length)	ggacatatgttctctactgctgttgc	cgtgctcgagaccgatgacttgatagc	58°C	This study
**Site mutagenesis**				
S120T	acaggagagacttacactggtggacac	tccagagccttgagttggtgc	60°C	This study
Y122F	agtctctccggatccagagc	tttactggtggacacggaacc	60°C	This study
G129A	ggctactccgttttgataaaagtaggtt	gcagcctgcggtaccgtg	60°C	This study
C141A	ggcaccggctactccg	gcaggtaccgtgcattcggacag	60°C	This study

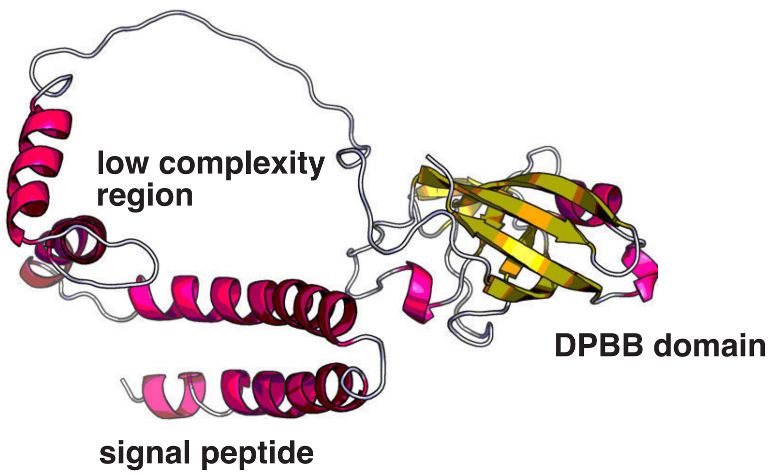

Figure S1. The RsRplA 3D structure as predicted by the RaptorX server. Yellow arrows indicate β strands and red areas indicate α helices

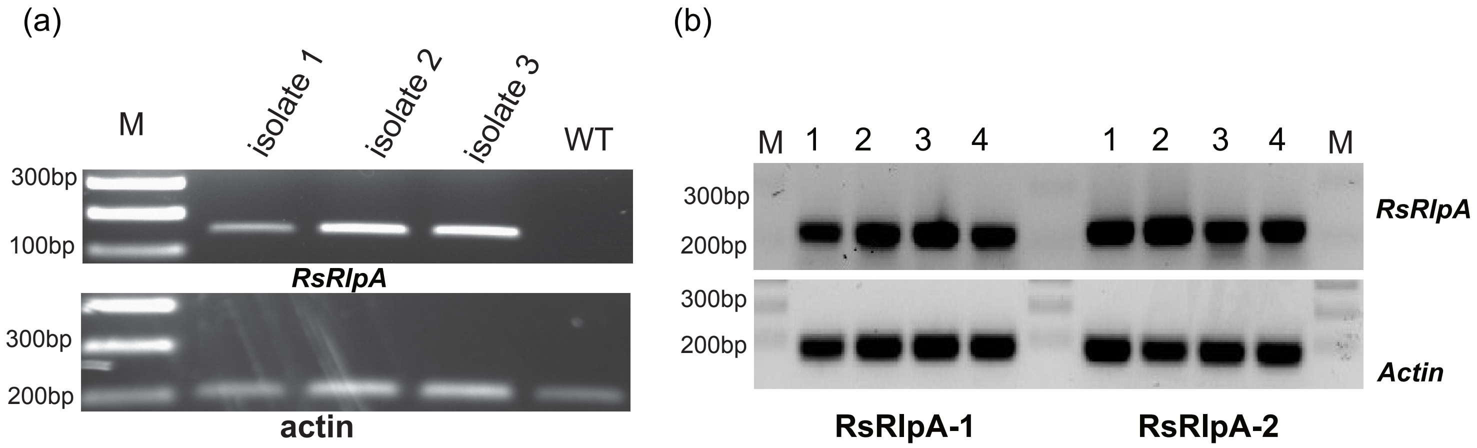

Figure S2. Validation of overexpression of the *RsRlpA* gene in *C. beticola* and *A. thaliana.* (a) RT-PCR on *C. beticola*.Transcripts from cDNA extracted from mycelia grown on PDA medium, for 7 days*. Cercospora beticola* wild type (WT) was used as negative control and *act* gene used as a positive control. (b) RT- PCR on *A. thaliana* overexpression lines. Expression of actin gene was used as a control. Two independent lines (RsRlpA-1 and RsRlpA-2) were used including four biological replicates (1-4). (M) Gene ruler.

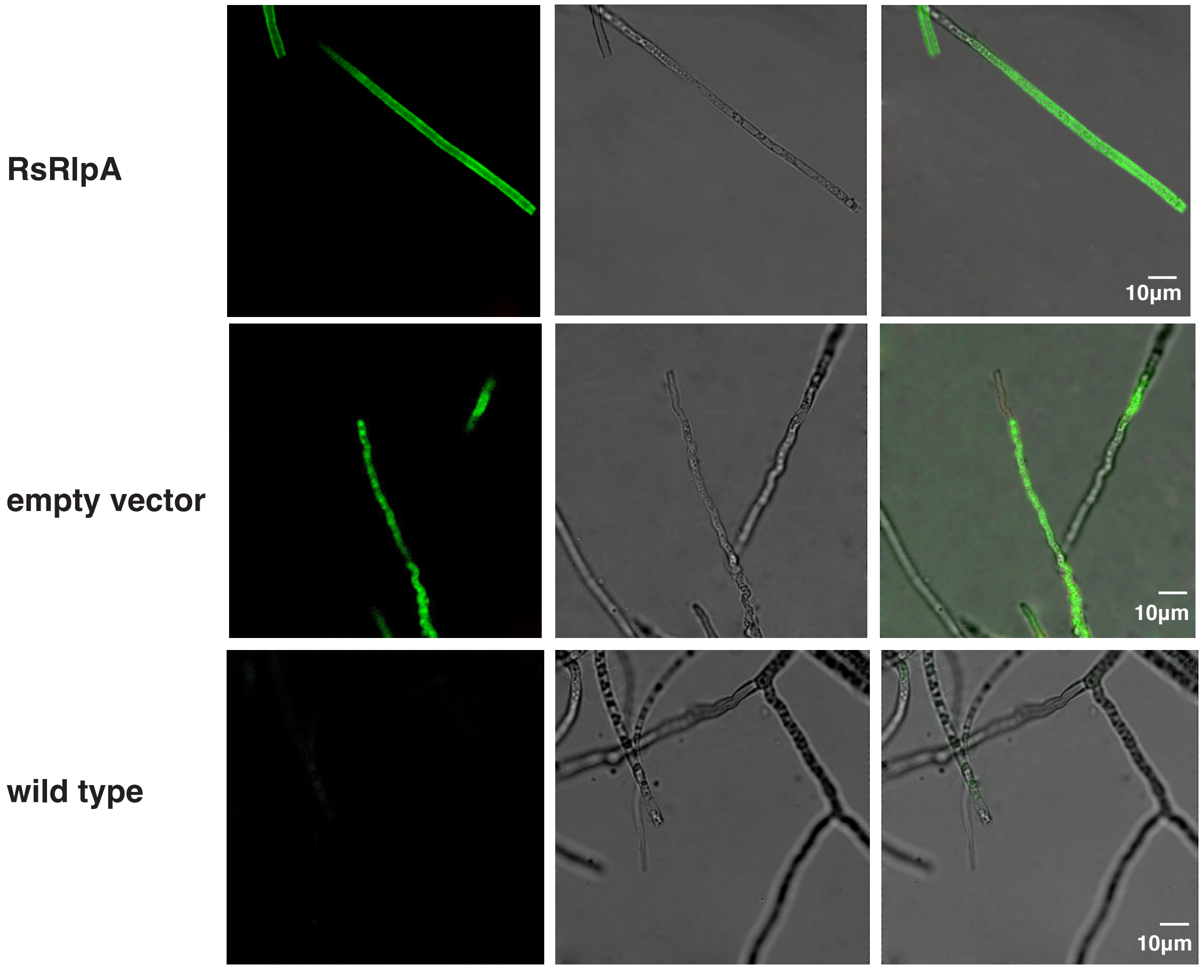

Figure S3. Live-cell imaging of GFP-tagged RsRlpA overexpressed in *C. beticola*. Live-cell imaging was performed with a laser-scanning confocal microscope with a sequential scanning mode and was excited at 488 nm and collected at 505–525 nm.

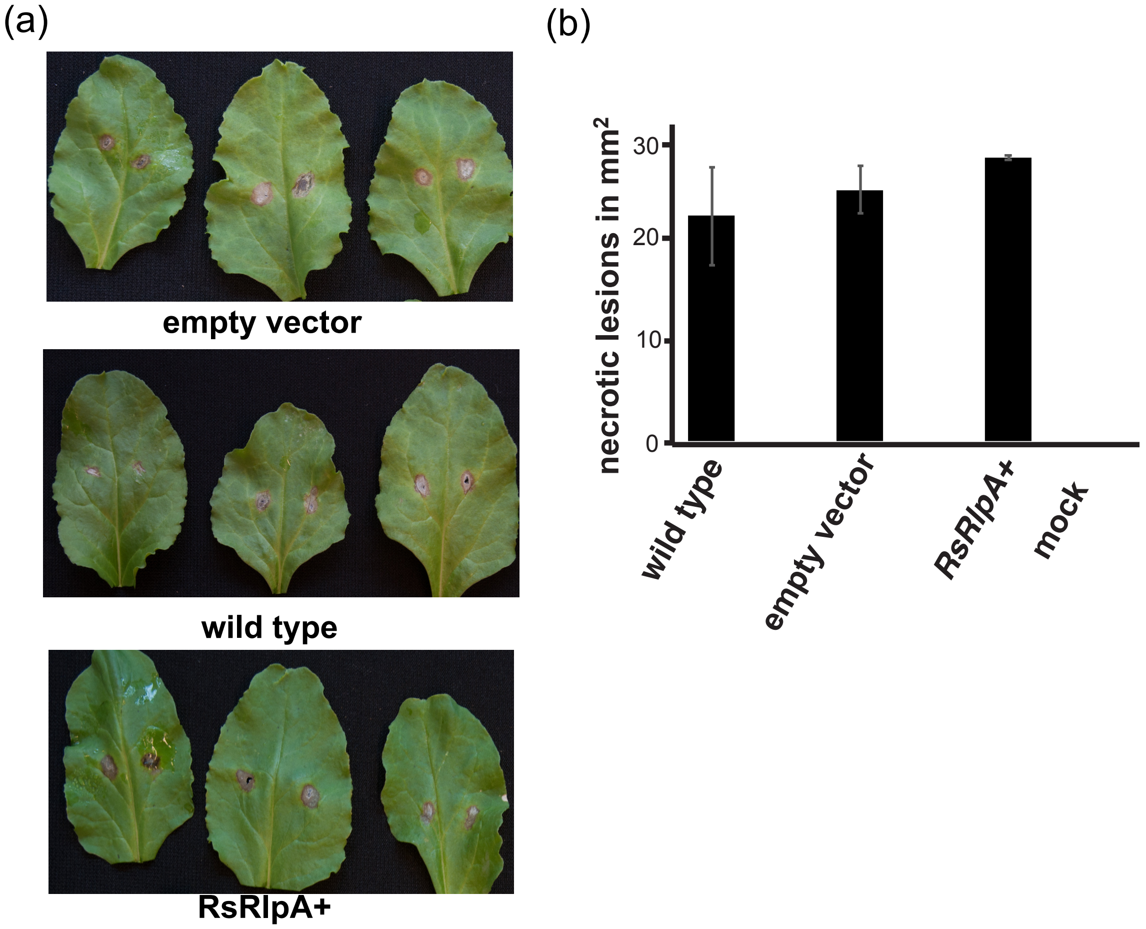

Figure S4. Overexpression of the *RsRlpA* gene in *Cercospora beticola* did not enhance necrosis. (a) Symptoms on sugar beet leaves in response to *C. beticola* strains harboring the *RsRplA* gene (*RsRplA*+) driven by the gdpA promoter, 7dpi. (b) Area of necrotic lesions on sugar beet leaves. Data show the average of three independent strains and each set includes three biological replicates. Error bars represent standard deviation. No statistical significant difference were observed among the treatments (WT, empty vector and *RsRplA*+) according to Tukey's test ($p < 0.05$)

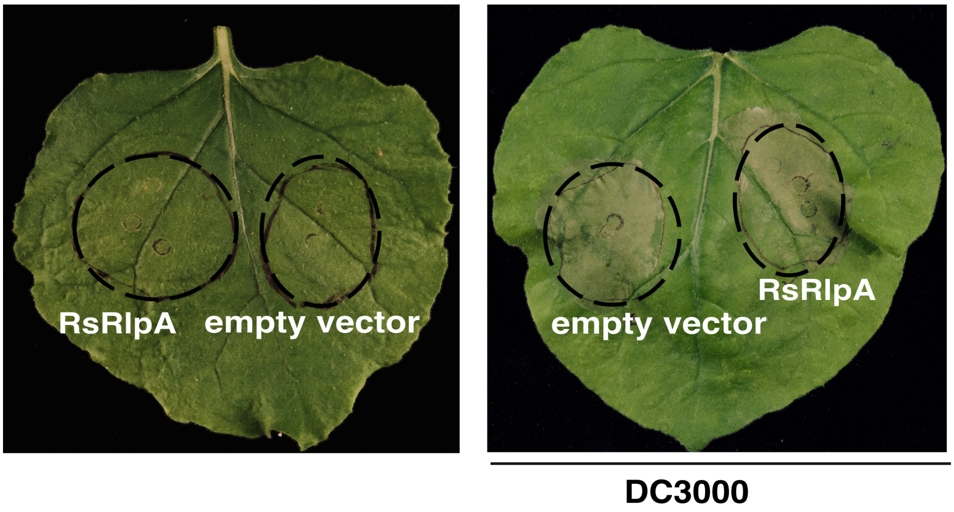

Figure S5.The RsRplA effector cannot suppress HR induced by *Pseudomonas syringae* pv *tomato* DC3000 strain. Agro-infiltration with only the RsRplA protein or the empty vector did not cause any symptom in *N. betathamiana* plants. Leaves were Agro-infiltrated first with the RsRlpA effector driven by the 35S promoter and HR challenged 24hpi with the bacterial strain.
